# Supplementary figures and images for: Predicting the functional impact of KCNQ1 variants with artificial neural networks
Source: PLoS Comput Biol. 2022 Apr 20;18(4):e1010038. doi: 10.1371/journal.pcbi.1010038 (PMC9060377; doi:10.1371/journal.pcbi.1010038)

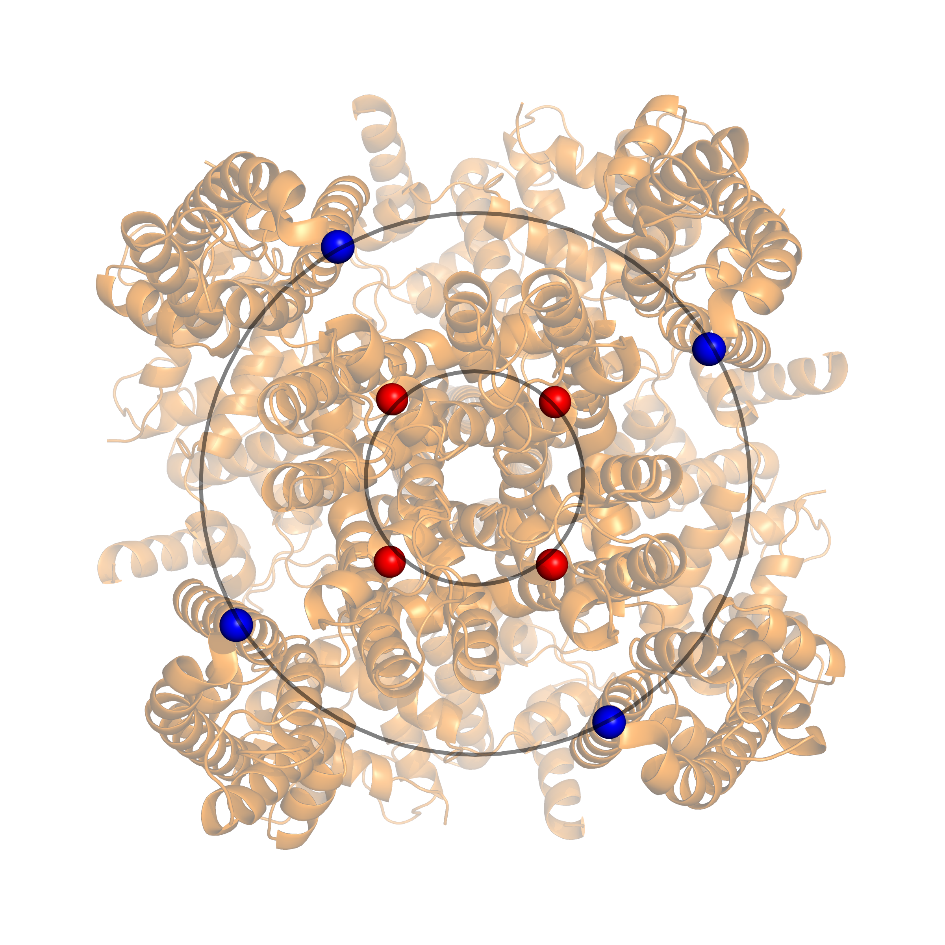

Supplement: S1 Fig — (DOCX) [file pcbi.1010038.s001.docx]

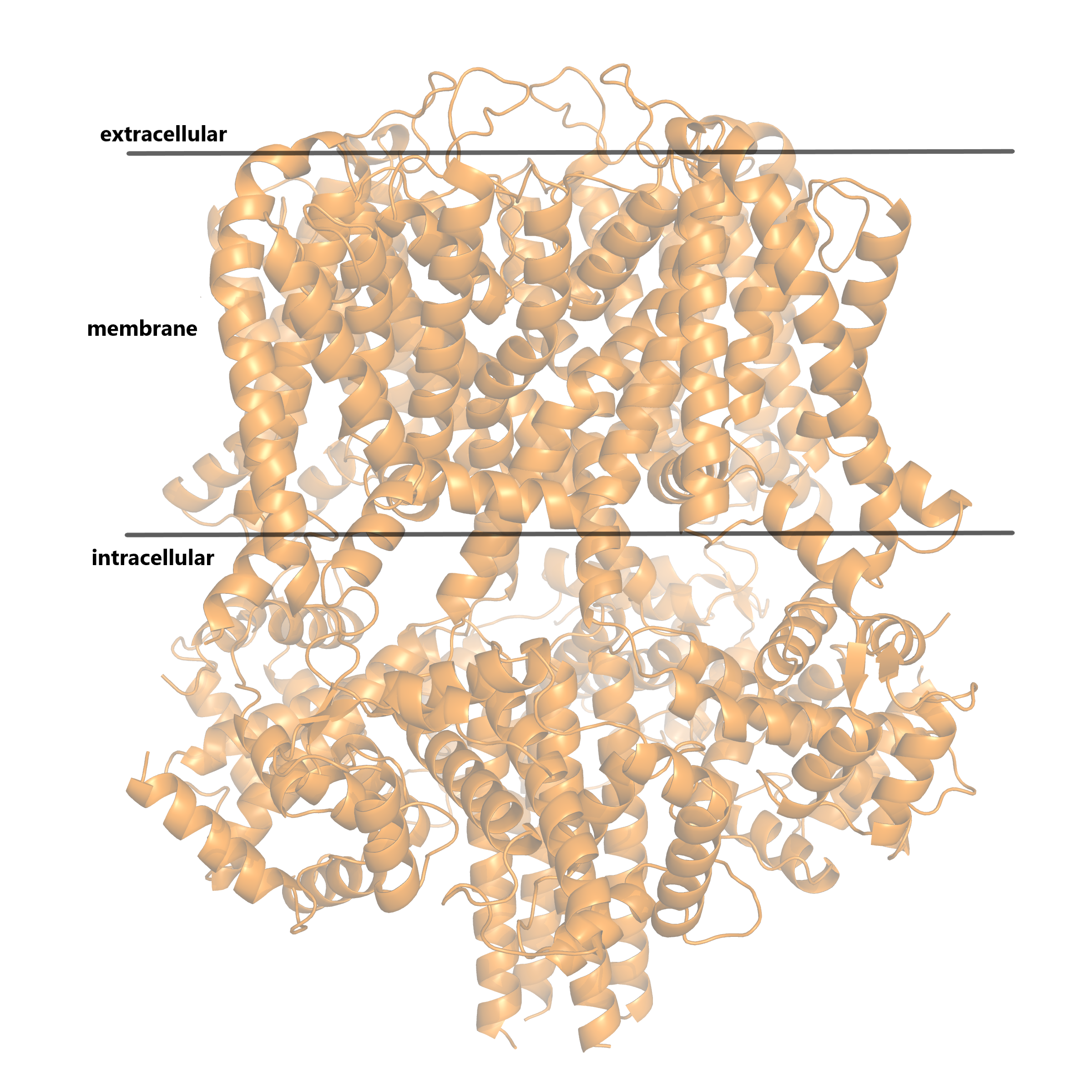

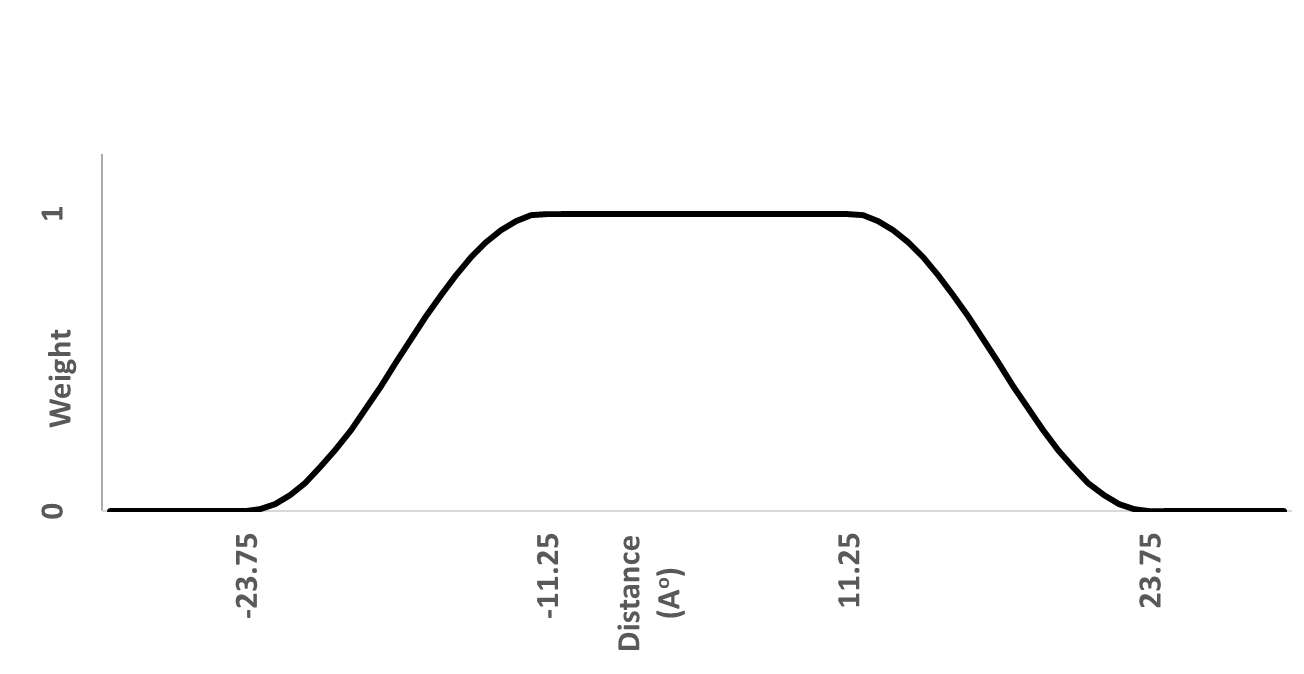

Supplement: S2 Fig — (DOCX) [file pcbi.1010038.s002.docx]

Solution

Interface

Membrane


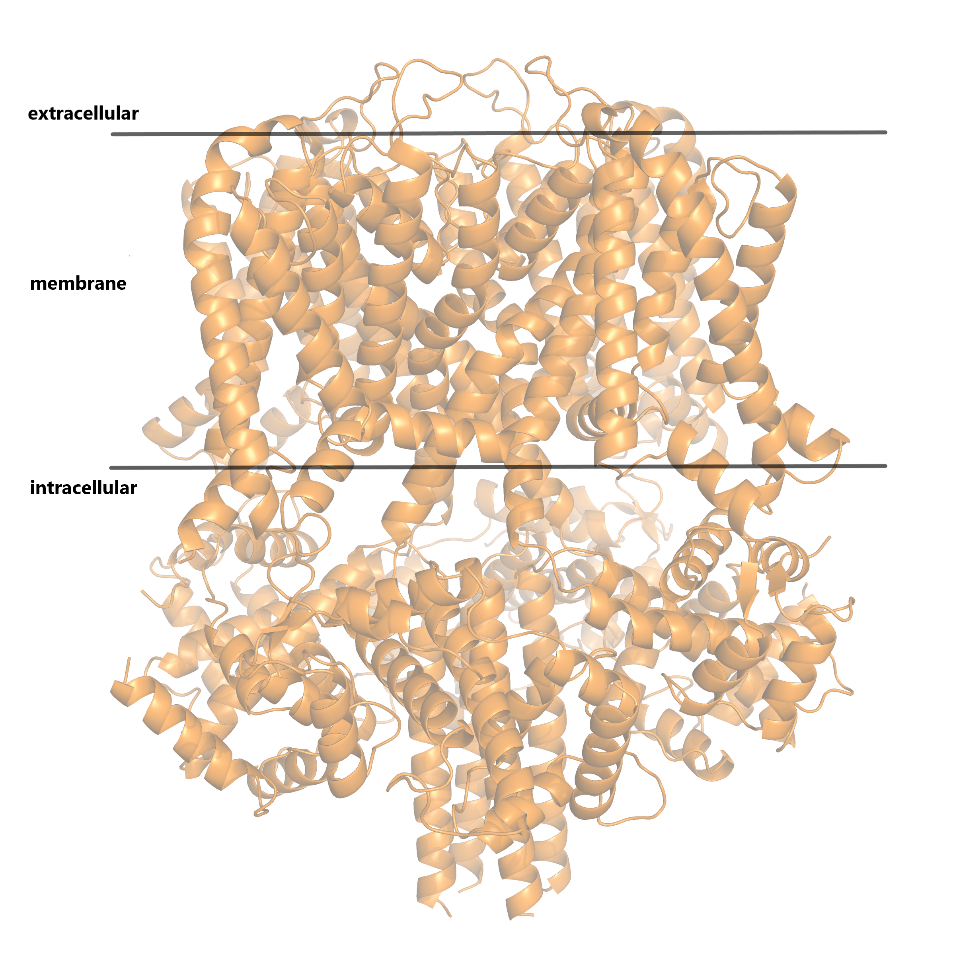

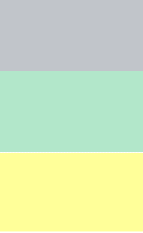

Supplement: S3 Fig — (DOCX) [file pcbi.1010038.s003.docx]

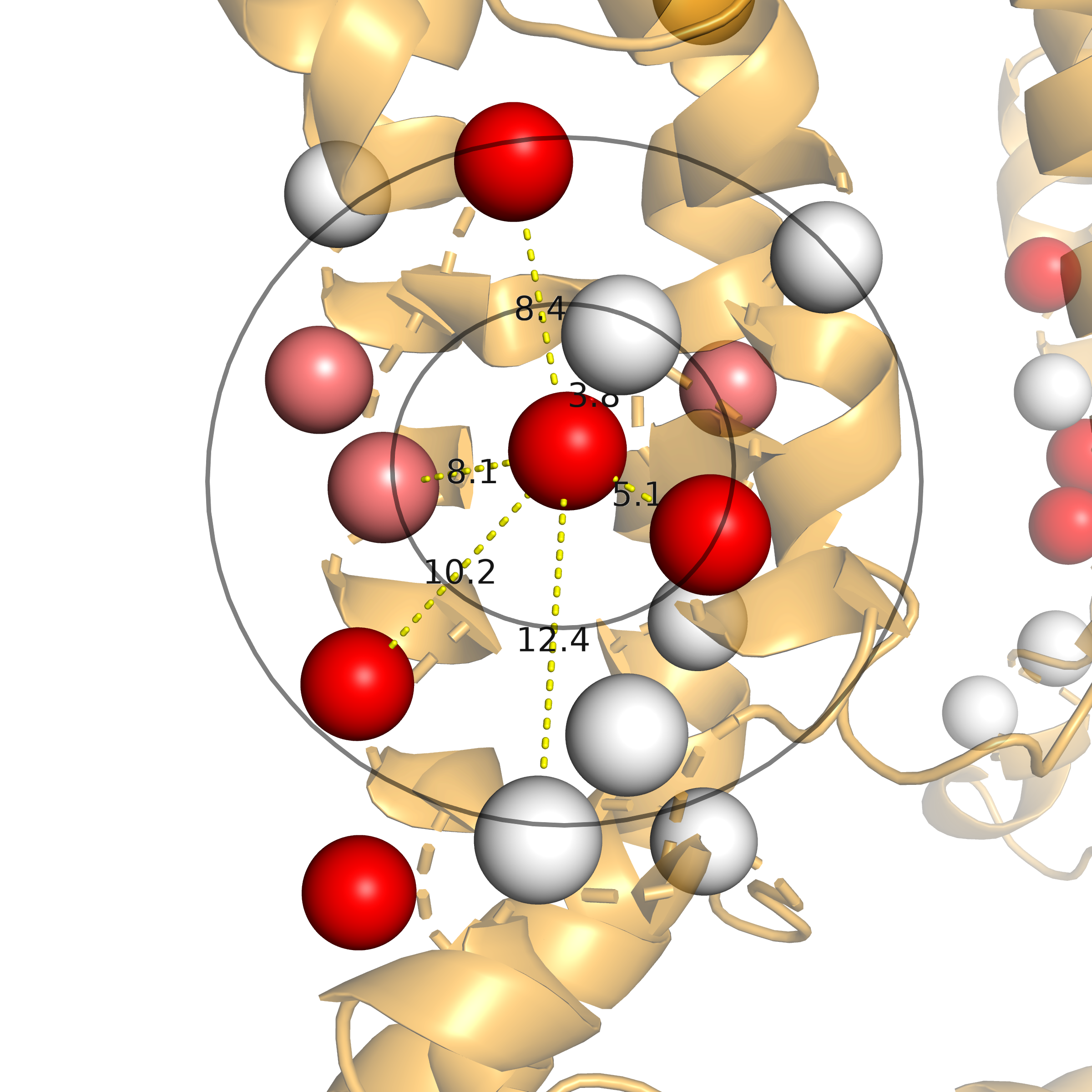

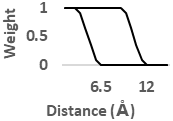

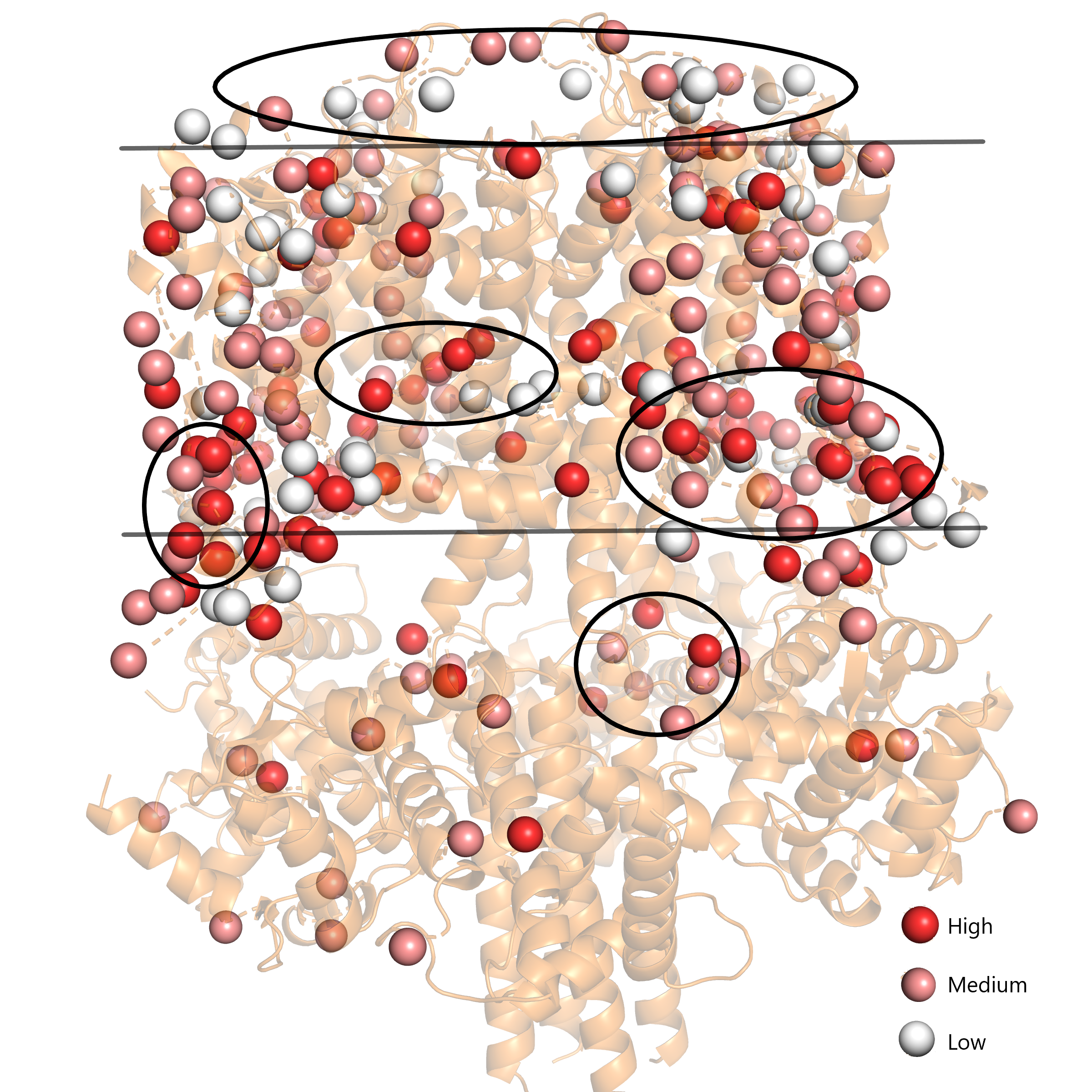

Supplement: S4 Fig — This also captures the concept of functional density by quantifying these clusters of polarizabilities for different neighborhood size. (DOCX) [file pcbi.1010038.s004.docx]

Polarizability


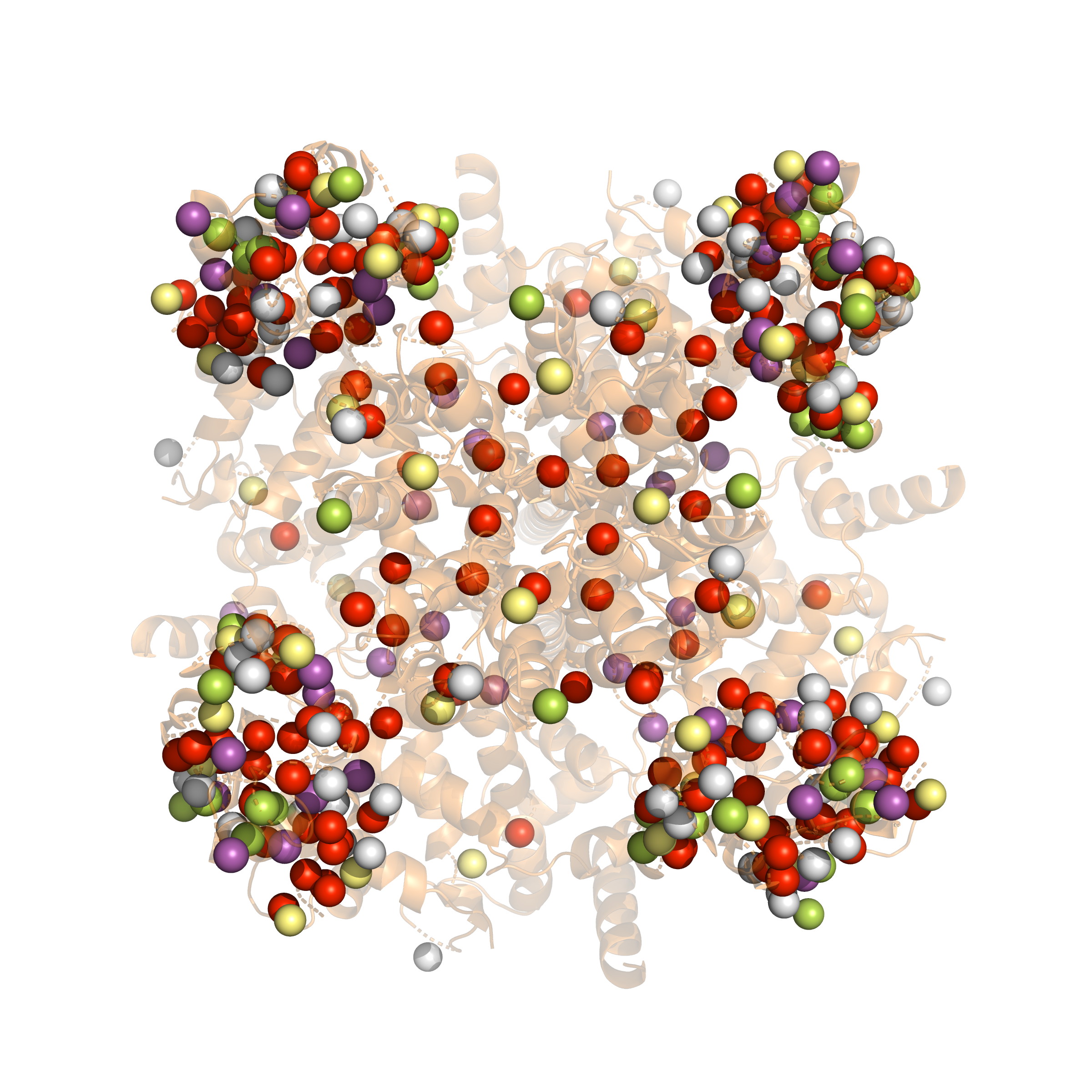

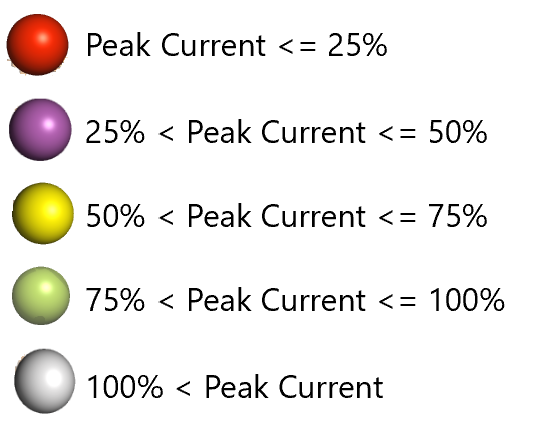

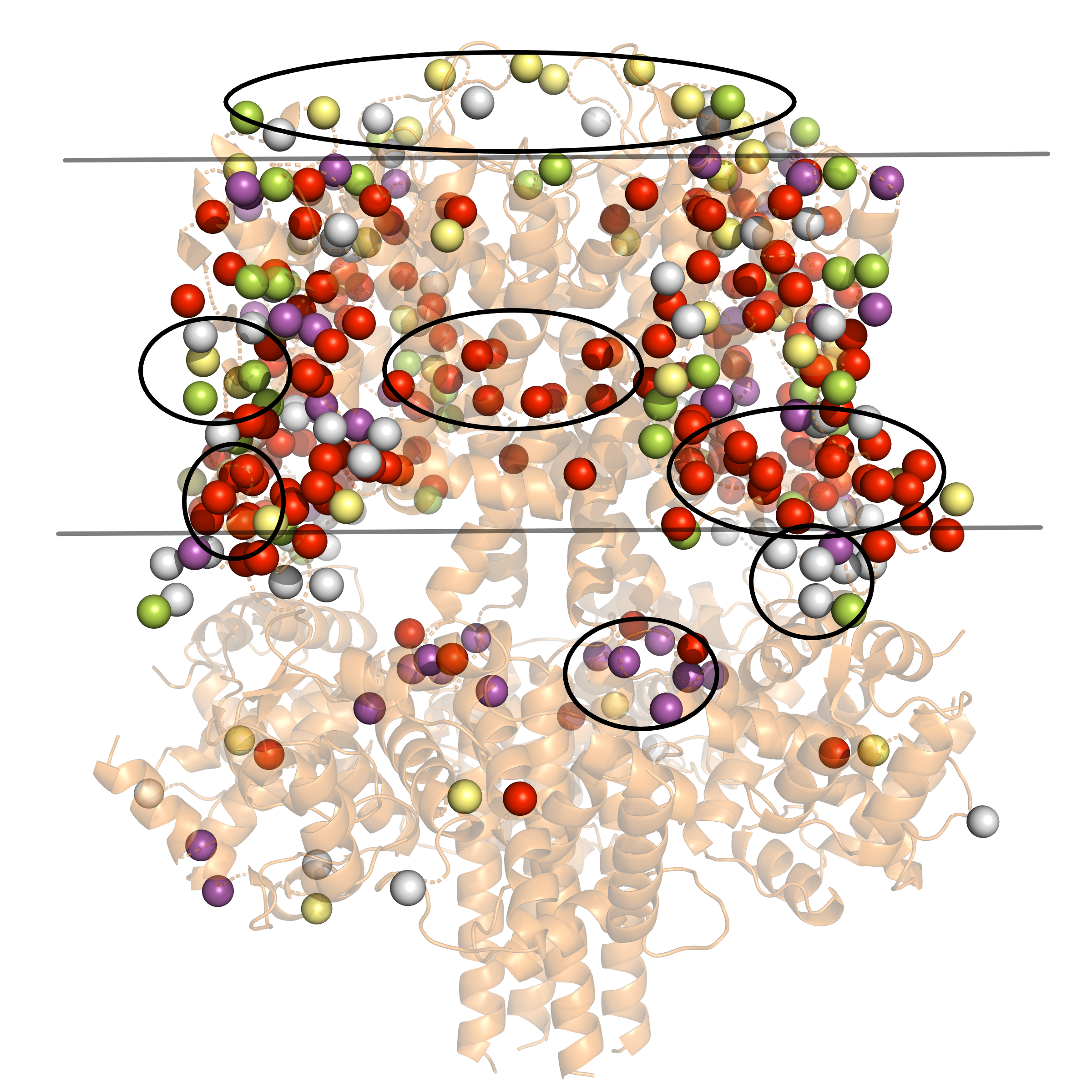

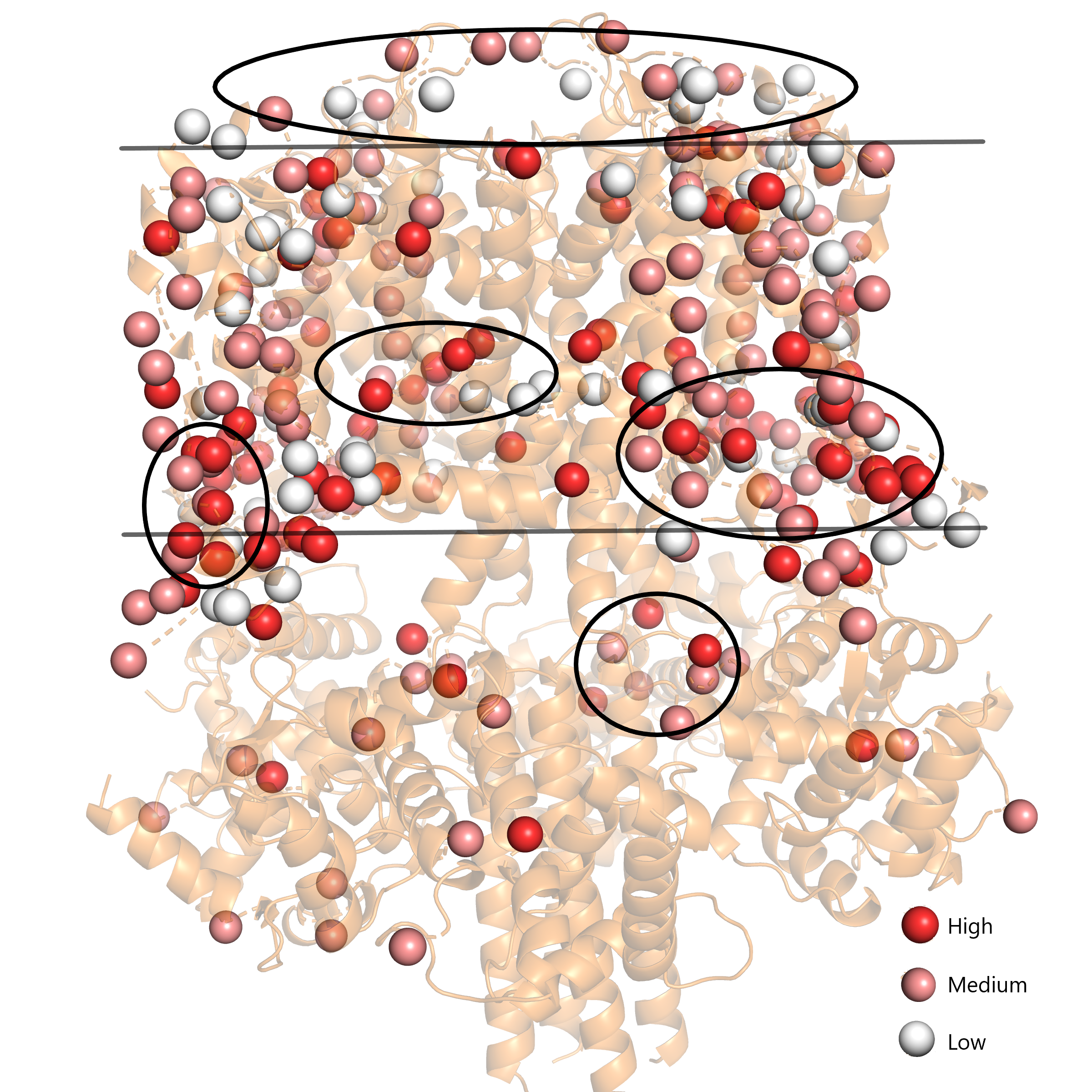

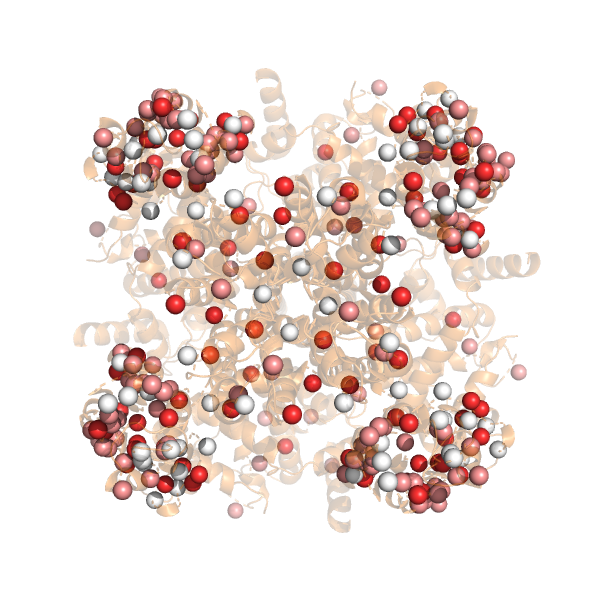


Side View

Top View

Supplement: S5 Fig — (DOCX) [file pcbi.1010038.s005.docx]

**
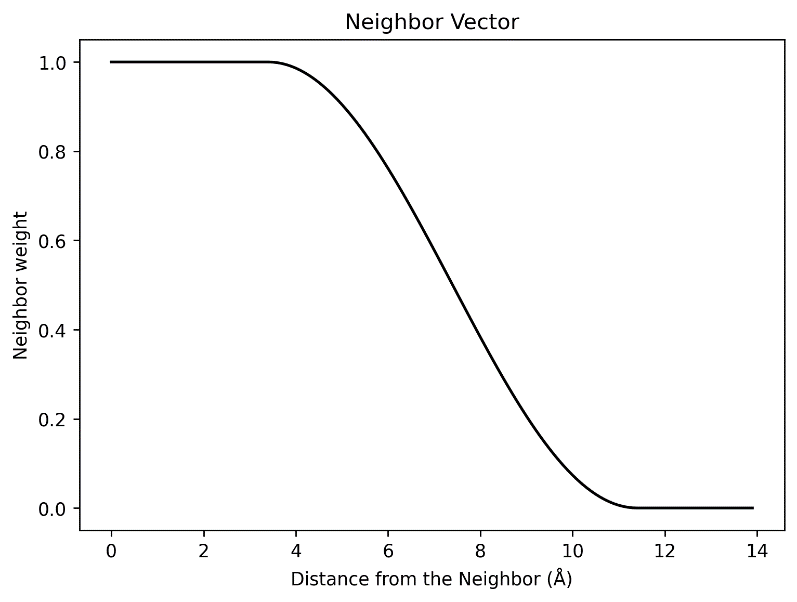
**

Supplement: S6 Fig — (DOCX) [file pcbi.1010038.s006.docx]

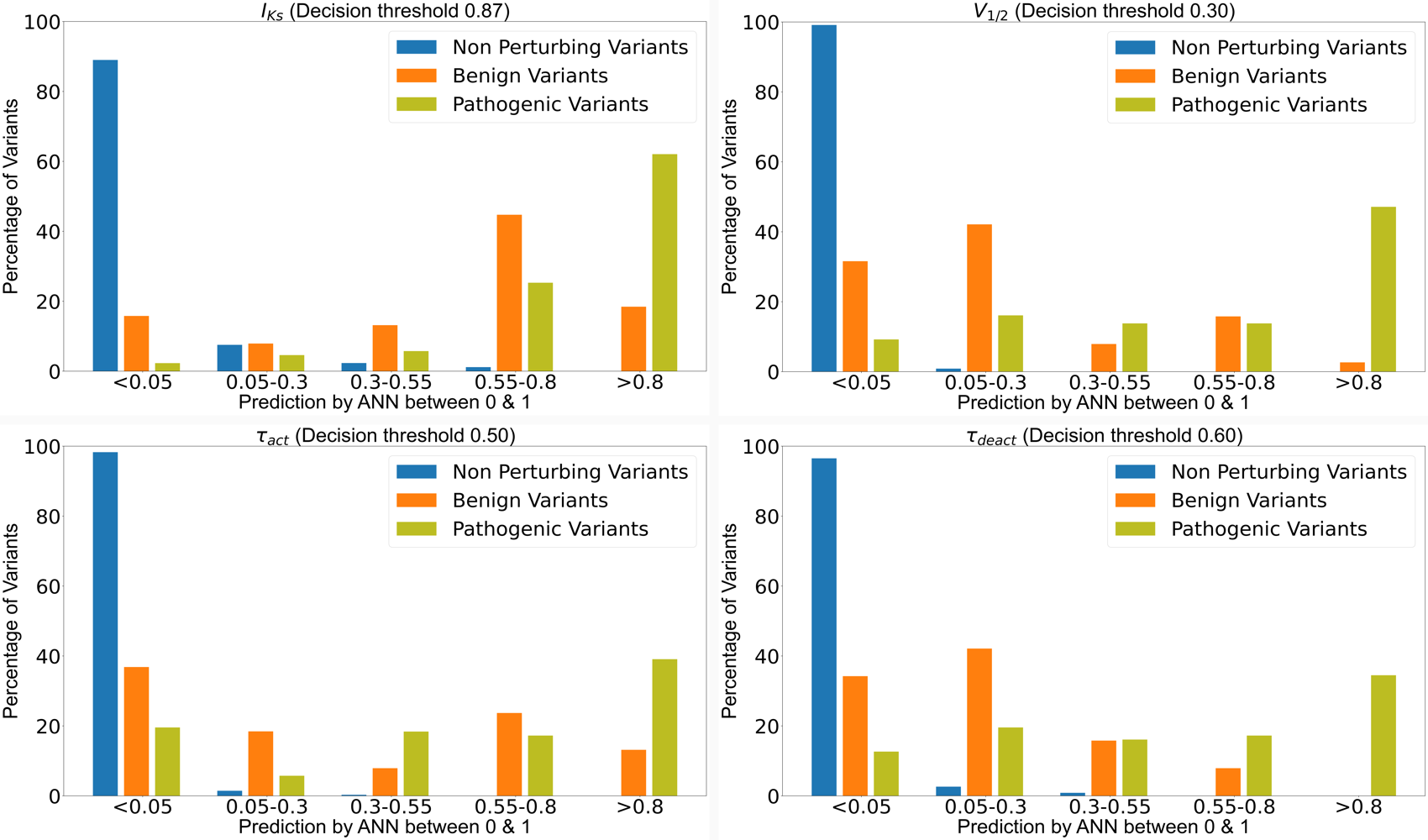

Supplement: S7 Fig — Decision threshold is between benign and pathogenic variants. (DOCX) [file pcbi.1010038.s007.docx]

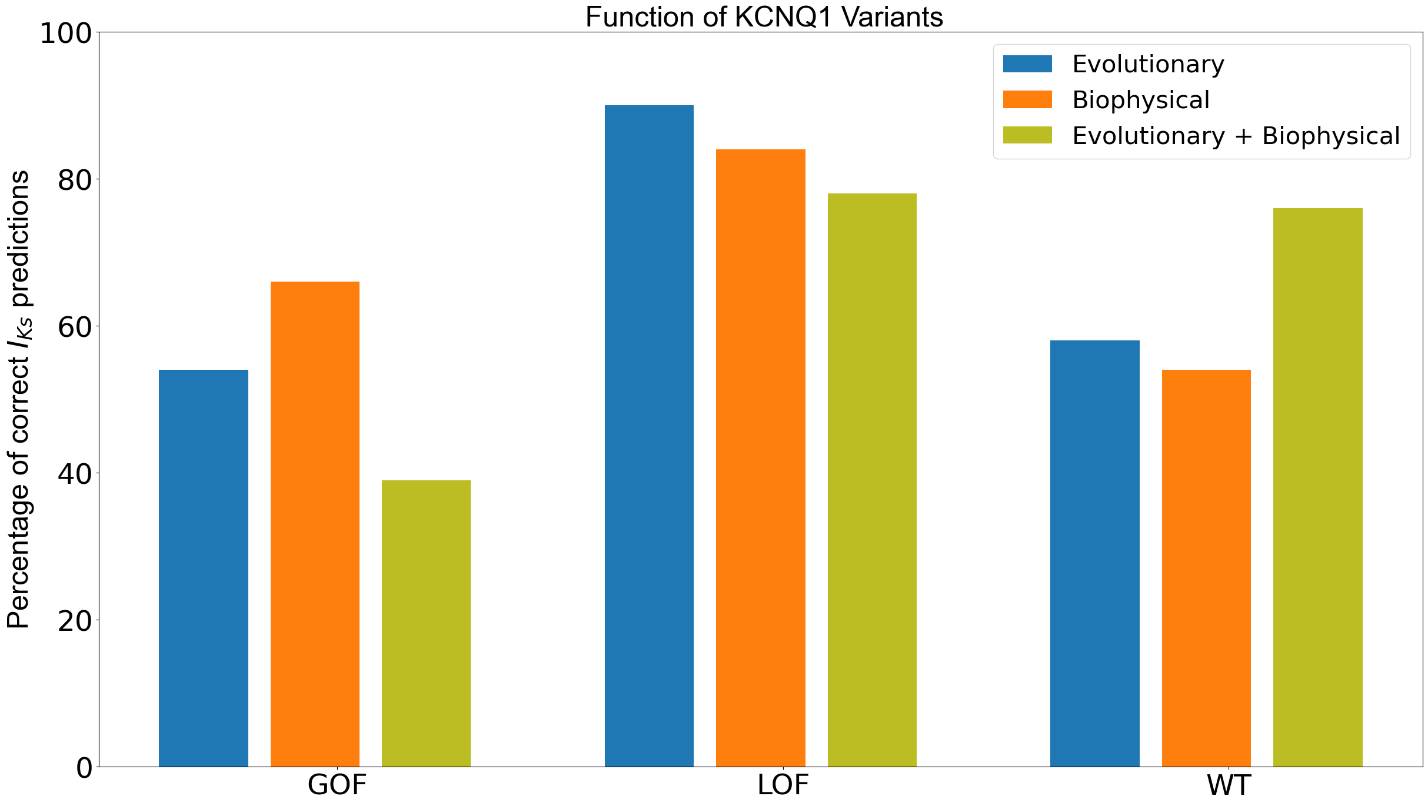

Supplement: S8 Fig — (DOCX) [file pcbi.1010038.s008.docx]

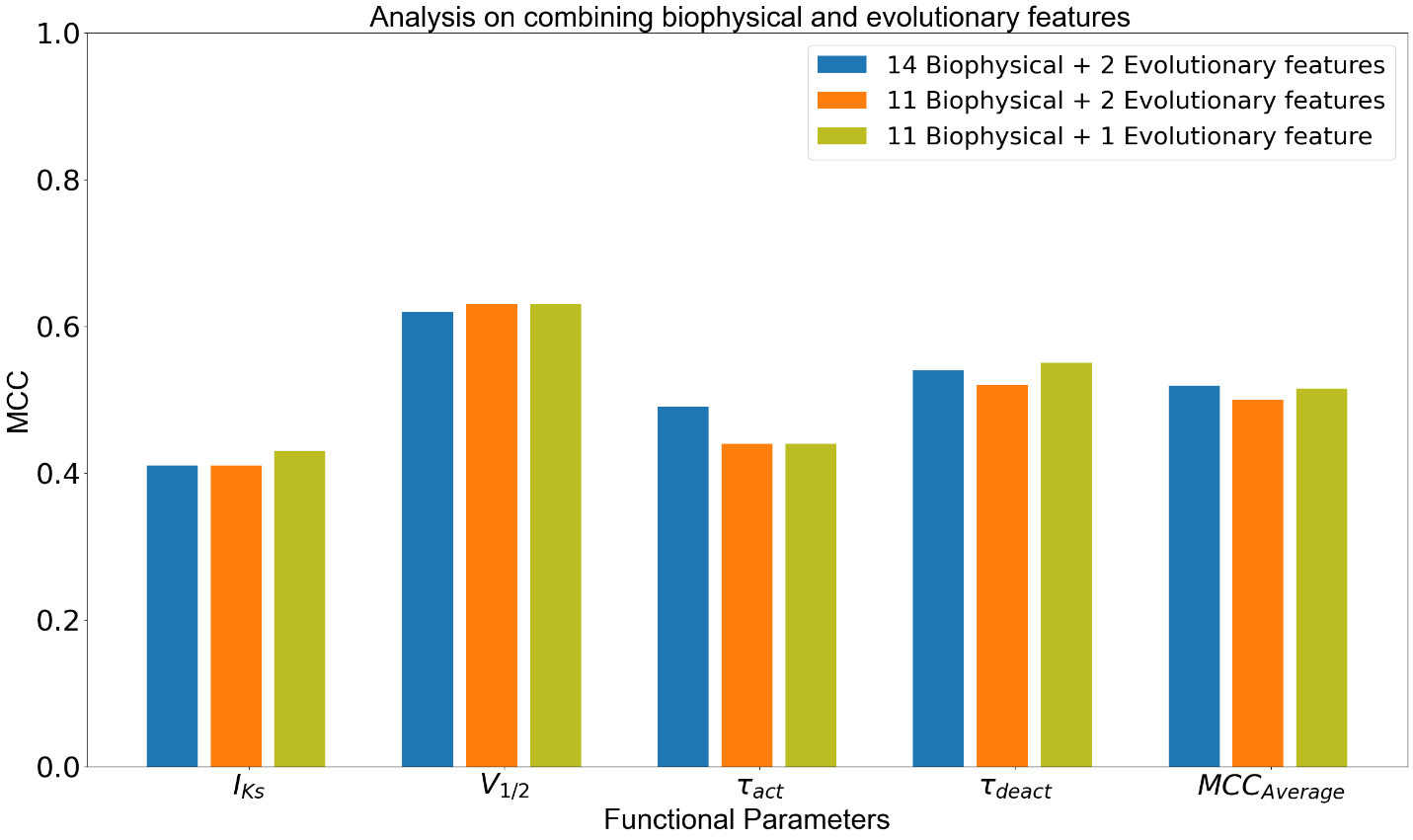

Supplement: S9 Fig — (DOCX) [file pcbi.1010038.s009.docx]
