# Supplementary material for: Predicting the functional impact of KCNQ1 variants with artificial neural networks
Source: PLoS Comput Biol. 2022 Apr 20;18(4):e1010038. doi: 10.1371/journal.pcbi.1010038 (PMC9060377; doi:10.1371/journal.pcbi.1010038)
Supplement: S1 Protocol Capture — (DOCX) [file pcbi.1010038.s011.docx]

**Predicting the Functional Impact of KCNQ1 Variants with Artificial Neural Networks**

Saksham Phul^1,2^, Georg Kuenze^1,2,3^, Carlos G. Vanoye^4^, Charles R. Sanders^1,5^, Alfred L. George, Jr.^4^, Jens Meiler^1,2,3,6,*^

^1^Center for Structural Biology, Vanderbilt University, Nashville, Tennessee, USA

^2^Department of Chemistry, Vanderbilt University, Nashville, Tennessee, USA

^3^Institute for Drug Discovery, Leipzig University, Leipzig, Saxony, Germany

^4^Department of Pharmacology, Northwestern University Feinberg School of Medicine, Chicago, Illinois, USA

^5^Department of Biochemistry, Vanderbilt University, Nashville, Tennessee, USA

^6^Department of Pharmacology, Vanderbilt University, Nashville, Tennessee, USA

*[jens@meilerlab.org](mailto:jens@meilerlab.org)

**Protocol Capture**

Table of content

1) Features generation…………………………………………………………………………….3

2) Model Training…………………………………………………………………….…………..4

3) Performance analysis……………………………………………………………….………….7

4) Input sensitivity analysis…………………………………………………….…………………9

1. *Features Generation*

All the models for this work were developed using Bio Chemical library (BCL). Before an ANN model is trained in BCL, we need to prepare a bin file that consists of descriptors and result labels. The following command line below generates a bin file that BCL can read for the purpose of training.

bcl-apps-static.exe GenerateDataset \

-source "ProteinMutationsDirectory(./, key file=./keys.txt, aa class=AAComplete, mutation extension=.data_file.csv, suffix=.pdb, add self mutation fraction=0.0)" \

-output <output_bin_file_for_model.bin> \

-feature_labels <feature_file.obj> \

-result_labels <resultslabel_file.obj> \

-id_labels 'Combine (MutationId)'

The input required for the above command line are:-

1. keys.txt file that contains name of the protein structure file
2. a protein structure in pdb format named as mentioned in keys.txt
3. file named as .uniref50.0.00001.ascii4 and .nr.ascii4 contains position specific scoring matrix from two different databases. These files are read through features_file.obj file
4. a data file that contains that variant IDs with functional data.

The command line computes biophysical features from the protein structure and evolutionary data from NCBI non-redundant database and uniref50 database. The *feature_file.obj* is the file that instructs the BCL program which features shall be computed. The *result_file.obj* file instructs the BCL program which labels shall be extracted from data csv file and passed to the model to train against these labels.

Position specific scoring matrix can be generated using psiblast.exe with following command line

psiblast -db <Name of the database> -query <protein sequence in fasta format> -num_iterations <No. of iterations> -inclusion_ethresh <hit threshold> -evalue <hit threshold> -num_alignments 0 -out_pssm <Output file name> -comp_based_stats 0 -use_sw_tback >> output.log 2>&1

1. *Model Training*

Once we have generated a bin file, an ANN model can be trained using the following BCL command :-

bcl-apps-static.exe model: Train \

"NeuralNetwork (

balance=True, \

balance target ratio=1, \

balance max repeats=100, \

transfer function = Rectifier (0.05), \

weight update = Simple(alpha=<momentum>, eta=<learning rate>), \

input dropout type=Zero, \

objective function =Accuracy(cutoff=0.5), \

input noise=0.0, \

iteration weight update=Attenuate(0.0,0.0,0.0,0.001), \

shuffle=True, \

steps per update=1, \

dropout(<input_layer_dropout>, <first hidden layer dropout>, <second hidden layer dropout>), \

hidden architecture (<No._of_ neurons_in_first_layer, No._of_ neurons_in_second_layer),

rescale output dynamic range=True,rmsd report frequency=1, scaling=AveStd)"

-max_minutes <number minutes to train>

-max_iterations <No. of iterations>

-opencl Disable

--result_averaging_window 0

-final_objective_function <objective function for training>

-feature_labels <features_file.obj>

-result_labels <results_file.obj>

-id_labels '<id_labels>'

-training "<training_data>"

-monitoring "<validation_data>"

-independent "<prediction_data>"

-print_independent_predictions “<storage predicted result to>”

-storage_model "File (directory=<storage model address>, prefix=model, key=<Model ID>)"

This command line trains a single model with fixed training, validation, and independent dataset. Here, architecture of ANN can be specified using hidden architecture tag. weight update tag sets learning rate and momentum for the network. Using -training, -monitoring, and -independent tags, training data, validation data, and independent dataset respectively can be passed to model to validate model performance. For this work, we adopted a 25-fold cross validation technique to remove any biasness and have better generalizability while making the predictions. A slurm script *(script_chunk.s*h) assisted in validation by dividing the dataset into 25 subsets. This script requires 25 threads that run the calculation on 25 different independent subsets simultaneously. Each thread runs over 25 validation datasets to predict a single independent dataset. The BCL command line below divides the dataset into 25 subsets wherein one subset was used as monitoring, one subset for independent testing, and rest for training.

Subset(filename=<output_bin_file_for_model.bin>, number chunks=<Number of subsets>, chunks=<Specify the subsets to use>)

We can alter chunks in above BCL command to specify subsets to use for -training , -monitoring , and -independent flags in training BCL command line. The for loop and if/else conditionals were used in bash *Script_chunk.sh* to vary the number of subsets for monitoring and validation.

---------------------------------------------------------Script_chunk.sh------------------------------------------------------------

#!/bin/bash

#SBATCH --nodes=1

#SBATCH --ntasks=1

#SBATCH --mem-per-cpu=2G

#SBATCH --time=0:50:00

#SBATCH --array=0-24

#SBATCH --job-name="Q1PredVarBio"

no_monitoring_set=24

divide=25

# first hidden layer size

netsize="32"

# second hidden layer size

netsize2="12"

# input layer dropout

visdrop="05"

# first hidden layer dropout

hdrop="33"

hdrop2="33"

iteration=1200

learning=0.002

momem=0.5

input_file=a1q1.mutations.combined155123.csv

features=features_combined.obj

results=resultslqts_combined.obj

MODEL_ID=${SLURM_ARRAY_TASK_ID}

#this loop runs over the monitoring sets and model ID runs over the independent sets

for a in $(seq 0 $no_monitoring_set);

do

if [ $a -ne $MODEL_ID ];

then

MONIND="Subset (filename= <output_bin_file_for_model>, number chunks=$divide, chunks=[$a])"

TRAIN="Subset(filename=full.whet.multimer_combined.bin,number chunks=$divide,chunks=[0]+[1]+[2]+[3]+[4]+[5]+[6]+[7]+[8]+[9]+[10]+[11]+[12]+[13]+[14]+[15]+[16]+[17]+[18]+[19]+[20]+[21]+[22]+[23]+[24]-[$a]-[$MODEL_ID])"

else #boundary cases

if [ ${a} == 0 ];

then

MONIND="Subset (filename= <output_bin_file_for_model>, number chunks=$divide, chunks=[1])"

TRAIN="Subset(filename=full.whet.multimer_combined.bin,number chunks=$divide,chunks=[0]+[1]+[2]+[3]+[4]+[5]+[6]+[7]+[8]+[9]+[10]+[11]+[12]+[13]+[14]+[15]+[16]+[17]+[18]+[19]+[20]+[21]+[22]+[23]+[24]-[1]-[$MODEL_ID])"

else

b=$(($a-1))

MONIND="Subset (filename=full.whet.multimer_combined.bin,number chunks=$divide, chunks=[$b])"

TRAIN="Subset(filename=full.whet.multimer_combined.bin,number chunks=$divide,chunks=[0]+[1]+[2]+[3]+[4]+[5]+[6]+[7]+[8]+[9]+[10]+[11]+[12]+[13]+[14]+[15]+[16]+[17]+[18]+[19]+[20]+[21]+[22]+[23]+[24]-[$b]-[$MODEL_ID])"

fi

fi

IND="Subset (filename=full.whet.multimer_combined.bin, number chunks=$divide, chunks=[$MODEL_ID])"

IDN=$1

rm -f models/$IDN/model`printf %06d $MODEL_ID`.*

#BCL training command line

bcl-apps-static.exe model:Train "NeuralNetwork(balance=True, balance target ratio=1,balance max repeats=100,transfer function = Rectifier(0.05),weight update = Simple(alpha=$momem, eta=$learning),input dropout type=Zero, objective function =Accuracy(cutoff=0.5),input noise=0.0,iteration weight update=Attenuate(0.0,0.0,0.0,0.001),shuffle=True, steps per update=1,dropout(0.$visdrop,0.$hdrop,0.$hdrop2),hidden architecture($netsize,$netsize2),rescale output dynamic range=True, rmsd report frequency=1,scaling=AveStd)" -max_minutes 300 -max_iterations $iteration -opencl Disable --result_averaging_window 0 -final_objective_function 'Accuracy(cutoff=0.5)' -feature_labels $features -result_labels $results -id_labels 'Combine(MutationId)' -training "$TRAIN" -monitoring "$MONIND" -independent "$IND" -print_independent_predictions ./results/$IDN/independent${a}_${MODEL_ID}_monitoring${MODEL_ID}_number${a}.gz -storage_model "File(directory=models/$IDN/${a}/,prefix=model, key=$MODEL_ID)" > ./log_files/$IDN/independent${MODEL_ID}_monitoring${MODEL_ID}_number${a}.txt

cp results/$IDN/independent${a}_* results/$IDN/$a

done

exit 0

To better organize predictions by the model, we use run.sh script that creates separate folders – one for predictions obtained with every validation set. We can use run.sh directly to run this model. This script prepares the <output_bin_file_for_model> file and then, submits the script_chunks.sh to slurm scheduler. It also cleans up the folder with temporary files before the start of the *script_chunk.sh*. Command line to run the model conveniently is :

./run.sh <Name_of_model>

1. *Performance Analysis*

Once the predictions are made by the ANN model, we can change into result folder and compute model performance. In the result folder, there are 25 subfolders – one for every of the 25 different validation sets. In each subfolder, 25 prediction files exist that were created with different prediction datasets using same validation set. Here, we can concatenate the compressed output files into a single txt file with the command :-

zcat independent*.gz > concat.txt

This will output *concat.txt* file. First column of the concat.txt file has the variant id. Second column to fifth column have the prediction values for peak current (I_Ks_), voltage of half-maximal activation (V_1/2_), activation time τ_act_ and deactivation time τ_deact_ respectively. Sixth column to the last column have the functional labels (0 for normal and 1 for dysfunctional) used for training in the same order as predicted values. BCL recognizes this pattern and can directly evaluate the performance using the command line: -

bcl-apps-static.exe model:ComputeStatistics \

-input concat.txt \

-obj_function 'ContingencyMatrixMeasure (measure=MCC, parity=1, cutoff=0.5, adjustable cutoff=0)' 'ContingencyMatrixMeasure(measure=MCC, parity=1,cutoff=0.5,adjustable cutoff=1)' \

-filename_obj_function <final_result.txt>

This will generate *final_result.txt* that contained the average MCC for four prediction labels. This command will also output log messages to the screen that contain individual MCC and ROC values for the four prediction labels.

*Concat.txt* contains predictions for silent and missense mutations. Missense mutations can be separated from silent mutations using a python script *delete_silent.py*. The results reported in the paper were obtained considering only missense mutations.

To run this script

python delete_silent.py

---------------------------------delete_silent.py-----------------------------------------------

def reading_file(name):

a=[]

with open(name,"r") as f:

content=f.read().splitlines()

for i in range(0,len(content),1): # separates the missense mutations from silent mutations

if(content[i][0]!=content[i][4]):

a.append(content[i])

return a

def writing_file(filename,data):

with open(filename,"w") as f:

for p in range(0,len(data),1):

k=data[p].split(",")

d1=[k[0],k[1],k[2],k[3],k[4],k[5],k[6],k[7],k[8]]

d1=(str(d1).replace("[",""))

d1=(str(d1).replace("]",""))

d1=(str(d1).replace("'",""))

f.write(str(d1))

f.write("\n")

f.close()

a1=reading_file("concat.txt")

writing_file("deleted_silent.txt",a1)

----------------------------------------------END---------------------------------------------------------------

Input to this python script is *concat.txt* that will output *deleted_silent.txt* file. We evaluate the performance of our model on only missense mutations using following command line:-

bcl-apps-static.exe model:ComputeStatistics \

-input deleted_silent.txt \

-obj_function 'ContingencyMatrixMeasure (measure=MCC, parity=1, cutoff=0.5, adjustable cutoff=0)' 'ContingencyMatrixMeasure(measure=MCC, parity=1,cutoff=0.5,adjustable cutoff=1)' \

-filename_obj_function <final_result.txt>

1. *Input Sensitivity Analysis*

The consistency method can be implemented using command line :-

bcl-apps-static.exe descriptor:ScoreDataset

-source 'Subset(filename= <output_bin_file_for_model.bin>)'

-score "InputSensitivityNeuralNetwork(storage=File(directory=<address to model directory>, prefix=model),weights=ScoreDerivativeEnsemble(consistency=1))"

-output <output file containing input sensitivity >

This command line requires the path to BCL generated model files, output bin file, and name of the output file. The output file will have the input sensitivity for our model.
