# Supplementary material for: Predicting the functional impact of KCNQ1 variants with artificial neural networks
Source: PLoS Comput Biol. 2022 Apr 20;18(4):e1010038. doi: 10.1371/journal.pcbi.1010038 (PMC9060377; doi:10.1371/journal.pcbi.1010038)
Supplement: S1 Text — (DOCX) [file pcbi.1010038.s012.docx]

**Predicting the Functional Impact of KCNQ1 Variants with Artificial Neural Networks**

Saksham Phul^1,2^, Georg Kuenze^1,2,3^, Carlos G. Vanoye^4^, Charles R. Sanders^1,5^, Alfred L. George, Jr.^4^, Jens Meiler^1,2,3,6,*^

^1^Center for Structural Biology, Vanderbilt University, Nashville, Tennessee, USA

^2^Department of Chemistry, Vanderbilt University, Nashville, Tennessee, USA

^3^Institute for Drug Discovery, Leipzig University, Leipzig, Saxony, Germany

^4^Department of Pharmacology, Northwestern University Feinberg School of Medicine, Chicago, Illinois, USA

^5^Department of Biochemistry, Vanderbilt University, Nashville, Tennessee, USA

^6^Department of Pharmacology, Vanderbilt University, Nashville, Tennessee, USA

*[jens@meilerlab.org](mailto:jens@meilerlab.org)

**Supporting information**

S1 Fig: Distance from the channel pore axis

S2 Fig: Depth of the site of mutation on the membrane

S3 Fig: Definition of three regions of hydrophobicity utilized in this work

S4 Fig: This figure depicts polarizability distribution with clusters of high, medium, and low polarizability. This also captures the concept of functional density by quantifying these clusters of polarizabilities for different neighborhood size.

S5 Fig: Correlation of peak current with polarizability at different pockets in the protein structure

S6 Fig: A definition of neighbor that includes a smooth transition function used in the neighbor vector algorithm with lower bound at 3.3 Å and upper lower at 11.4 Å

S1 Table: Amino acid parameters

S7 Fig: Distribution of Prediction by ANN for non-perturbing, benign, and pathogenic variants depicting that ANN can distinguish these variants by predicting in three different regions between 0 and 1. Decision threshold is between benign and pathogenic variants

S8 Fig: Percentage of accurate predictions for GOF, LOF and WT-like based on peak current density by the three ANNs models considered in this study

S9 Fig: Exclusion of 3 biophysical features and 1 evolutionary feature does not affect the performance when evolutionary and biophysical features are combined

**Biophysical features**

*Distance from the channel pore axis*

The distance of mutation site from channel pore axis was an important biophysical feature that help identify functionality vital regions in the KCNQ1 Protein. The S1 Fig shows two different mutation sites with blue color site in VSD region and red color site in PD region.

*Burial of mutation site in the membrane*

The depth of the mutation site relative to membrane thickness was weighted based on three regions defined as mutation on the membrane, mutation at the protein solution interface, and mutation outside the membrane. The thickness of the membrane was considered as 31.4 Å. A decaying cosine function was used at the interface for a smooth transient from the site being on the membrane to outside the membrane as shown in S2 Fig. The criteria for depth of mutation are as follows:

$Depth\left( Distance\left( z \right) \right)=\left\{ \begin{aligned} 1, z<11.25 Å \\ \frac{1+\cos\left( \pi\times\frac{z-11.25}{23.75-11.25} \right)}{2}, 11.25 Å\leq z<23.75 Å \\ 0, z>23.75Å \end{aligned} \right.$ ………(1)

*Hydrophobicity an indicator of free energy change at the mutation site*

Hydrophobicity plays a vital role in the folding, structural stability, and functioning of the membrane protein. We utilized a previously developed hydrophobic scale for mammalian alpha helical membrane protein by Koehler *et al* [1]. Their work distinguishes amino acid preference for solution, interface, and membrane region as shown in S3 Fig. For consistency, we incorporated the same definition and thickness of interface and membrane. The value of hydrophobicity for an amino acid was taken based on the region where the site of the mutation exists.

*Functional density for polarizability and hydrophobicity*

In addition to structure-based feature discussed previously, we computed an average physiochemical property of the neighborhood around the site of mutation. The concept of functional density from Kroncke *et al* [2] quantifies the functional critical spots within the protein structure. This method is based on k-nearest neighbors’ algorithm, wherein average physiochemical property (for this study were polarizability and hydrophobicity) around the site of mutation was weighted by inverse of their distance from the site of mutation. For instance, functional density for polarizability is calculated as follows:

$$f\left( x,i \right)=\frac{\bar{Polarizability}+ \sum_{i}^{Variants} \left. Weight(Distance\left( x,i \right) \times Polarizability\left( i \right) \right)}{\left. 1+ \sum_{i}^{Variants} \left. Weight(Distance(x,i \right) \right)} \ldots.\ldots(2)$$

$$Weight\left( Distance\left( x,i \right) \right)=\left\{ \begin{aligned} & 1 Distance <X_{Start} \\ &\frac{1+\cos\left( \pi\times\frac{Distance\left( x,i \right)-X_{Start}}{X_{end}-X_{Start}} \right)}{2} X_{Start} \leq Distance <X_{End} .(3) \\ & 0 {Distance\geq X}_{End} \end{aligned} \right.$$

where $f(x,i)$ is the functional density at the site of interest with i^th^ neighbor residue within the threshold distance and to take prior knowledge into account, the average polarizability from the dataset was taken as pseudo count represented as $\bar{Polarizablity}$ in the equation 2. The summation runs for all the neighboring residue existing within the threshold distance from the residue of interest. A decaying cosine function of thickness 0.5 Å was used to have a smooth transition of weight towards the end of the neighbor threshold. For polarizability, we used two neighborhoods with different radius (X_End_) with first shell of contacting residues at 6.5 Å and a second shell at 12 Å (S4 Fig). We used similar implementation for hydrophobicity around the site of mutation except those two neighborhoods with different radius (X_End_) with first shell of contacting residues at 1 Å and a second shell at 6.5 Å. These radial distances are based on distance from C_ß_ of native amino acid.

*Neighbor vector captures buried site in the protein structure*

The sites that are buried within the core of the protein are highly conserved, critical to the stability, and functioning of the protein. Mutation at the conversed sites (or less exposed) can immensely impact the stability and functioning of the protein. Therefore, to identify these important sites from the protein structure, we recall neighbor vector definition from Durham *et al* [3]. Neighbor vector considers the spatial orientation of neighboring amino acids within a radius of 11.4 Å from the C_ß_ of the native amino acid. The magnitude of neighbor vector implies exposure of residue of interest in the protein environment wherein long lengths of this vector $\cong1$signify high exposure or otherwise.

*Amino acid parameters and physicochemical properties*

In addition to structure-based and environment-based features, we also introduce few physiochemical parameters of amino acid that improves the prediction. There properties were steric parameter, normalized vanderWaal volume, polarizability, number of hydrogen donor, and number of hydrogen acceptor sites [4]. These parameters are list in the Table 1 for 20 amino acids.

The steric parameter quantifies the complexity, symmetry, and branching at the $C_{\alpha}$ from the structure of amino acids [4]. The polarizability is related to molar refractivity as

$$\alpha=\frac{3}{4\pi N} .\frac{M}{d}.\frac{n^{2}-1}{n^{2}+2}$$

Where n is index of refraction, M is molecular weight , d is density, and N is number of atoms.

The normalized vanderWaal volume is defined as

$$v\left( side chain \right)=\frac{V\left( side chain \right)-V(H)}{V(CH_{2})}$$

This normalized volume is thus, 0 for glycine and 1 for alanine [4].

**Evolutionary features**

We used PSI blast technique to characterize the likelihood of an amino acid substitution from the perspective of protein evolution[5]. This technique was implemented by obtaining position specific scoring matrix (PSSM) by searching though uniref50 databases[6] and NCBI non-redundant sequence databases[7] with PSI-BLAST for four iterations. The E-value inclusion threshold was set to 0.00001. A PSSM matrix of size 676 x 20 gives log ratios of frequency of 20 amino acids to occur at 676 sites for KCNQ1 protein relative to the frequency of wild type amino acid.

$$Likehood of Amino acid substitution=\lambda ln\frac{P_{A}}{P_{A}^{o}}$$

where *P_A_* is the probability of amino acid A at a position,  *P^o^_A_* is the expected probability for wild type amino acid, and λ is a scaling factor built in PSI-BLAST[5].

The difference of PSSM score between mutant amino acid (A) and native amino acid (B) from two databases are as follows:

$$PSSM\left( NR \right)=\left( ln\frac{P_{A_{NR}}}{P_{A_{NR}}^{o}}-ln\frac{P_{B_{NR}}}{P_{B_{NR}}^{o}} \right)$$

$$PSSM\left( uniref50 \right)=\left( ln\frac{P_{A_{uniref50}}}{P_{A_{uniref50}}^{o}}-ln\frac{P_{B_{uniref50}}}{P_{B_{uniref50}}^{o}} \right)$$

PSSM(NR) and PSSM(uniref50) were evolutionary features used in this work that measure the perturbation due to amino acid substitution. Higher the perturbation, more likely it is to have a functional or structural impact on the protein due to mutation.

**Reference**

1. Koehler J, Woetzel N, Staritzbichler R, Sanders CR, Meiler J. A Unified Hydrophobicity Scale for Multi-Span Membrane Proteins. *Proteins* 2009; **76**: 13.

2. Kroncke BM, Duran AM, Mendenhall JL, Meiler J, Blume JD, Sanders CR. Documentation of an Imperative To Improve Methods for Predicting Membrane Protein Stability. *Biochemistry* 2016; **55**: 5002–5009.

3. Durham E, Dorr B, Woetzel N, Staritzbichler R, Meiler J. Solvent accessible surface area approximations for rapid and accurate protein structure prediction. *Journal of Molecular Modeling* 2009; **15**: 1093.

4. FAUCHÈRE J ‐L, CHARTON M, KIER LB, VERLOOP A, PLISKA V. Amino acid side chain parameters for correlation studies in biology and pharmacology. *International journal of peptide and protein research* 1988; **32**: 269–278.

5. Altschul SF, Madden TL, Schäffer AA, Zhang J, Zhang Z, Miller W *et al.* Gapped BLAST and PSI-BLAST: a new generation of protein database search programs. *Nucleic Acids Research* 1997; **25**: 3389–3402.

6. Suzek BE, Huang H, McGarvey P, Mazumder R, Wu CH. UniRef: comprehensive and non-redundant UniProt reference clusters. *Bioinformatics* 2007; **23**: 1282–1288.

7. Pruitt KD, Tatusova T, Maglott DR. NCBI reference sequences (RefSeq): a curated non-redundant sequence database of genomes, transcripts and proteins. *Nucleic Acids Research* 2007; **35**: D61.
