# Supplementary material for: Predicting the functional impact of KCNQ1 variants with artificial neural networks
Source: PLoS Comput Biol. 2022 Apr 20;18(4):e1010038. doi: 10.1371/journal.pcbi.1010038 (PMC9060377; doi:10.1371/journal.pcbi.1010038)
Supplement: S1 Table — (DOCX) [file pcbi.1010038.s013.docx]

| Amino acids | sTERIC pARAMETER | pOLARIZABILITY | vOLUME | nO. OF DONOR SITES | nO. OF ACCEPTOR SITES |
| --- | --- | --- | --- | --- | --- |
| ALA | 1.28 | 0.05 | 1.00 | 0 | 0 |
| GLY | 0.00 | 0.00 | 0.00 | 0 | 0 |
| VAL | 3.67 | 0.14 | 3.00 | 0 | 0 |
| LEU | 2.59 | 0.19 | 4.00 | 0 | 0 |
| ILE | 4.19 | 0.19 | 4.00 | 0 | 0 |
| PHE | 2.94 | 0.29 | 5.89 | 0 | 0 |
| TYR | 2.94 | 0.30 | 6.47 | 1 | 1 |
| TRp | 3.21 | 0.41 | 8.08 | 1 | 0 |
| THR | 3.03 | 0.11 | 2.60 | 1 | 1 |
| SER | 1.31 | 0.06 | 1.60 | 1 | 1 |
| ARG | 2.34 | 0.29 | 6.13 | 3 | 0 |
| LYS | 1.89 | 0.22 | 4.77 | 3 | 0 |
| HIS | 2.99 | 0.23 | 4.66 | 2 | 2 |
| ASP | 1.60 | 0.11 | 2.78 | 0 | 2 |
| GLU | 1.56 | 0.15 | 3.78 | 0 | 0 |
| ASN | 1.60 | 0.13 | 2.95 | 1 | 0 |
| GLN | 1.56 | 0.18 | 3.95 | 1 | 1 |
| MET | 2.35 | 0.22 | 4.43 | 0 | 0 |
| PRO | 2.67 | 0.00 | 2.72 | 0 | 0 |
| CYS | 1.77 | 0.13 | 2.43 | 0 | 0 |

S1 Table: Amino acid parameters
