# Supplementary material for: Predicting the functional impact of KCNQ1 variants with artificial neural networks
Source: PLoS Comput Biol. 2022 Apr 20;18(4):e1010038. doi: 10.1371/journal.pcbi.1010038 (PMC9060377; doi:10.1371/journal.pcbi.1010038)
Supplement: Attachment — Submitted filename: Response_to_reviewers.docx [file pcbi.1010038.s014.docx]

Response to the Reviewers:

We thank the reviewers for their helpful suggestions for revision, which led to an improved paper. Each comment is addressed below. To facilitate review, we include all changes applied to the manuscript below.

Reviewer #1

1) The authors state in the abstract that the functional predictions “can assist physicians in taking appropriate treatment decision for patients with genetic heart rhythm disorder.” This may well be true, but it’s hard to assess given the information in the paper. Importantly (as the authors are aware), clinical utility requires more than accurate predictions, including that they are actionable. I believe KCNQ1 is, but that is not clear from the paper. In addition, the prediction methods would need to be included in assigning variant effects, and again it is unclear whether the methods are better than what’s out there. Thus, while I don’t doubt that the methods developed are both interesting and could become useful, I would ask that the authors provide a bit more detail how these methods can actually help in treatment or remove this from the abstract. Although only a wording issue, I have added this in “major comments” section as I think it is important.

We acknowledge the concern raised by the reviewer. Thus, we removed the statement and toned down our conclusion by replacing it with Lines 14-18 as:

Page 1, Lines 14-18:

“Recent advances in experimental and computational protein structure determination have provided access to high-quality structures for most human proteins and mutants thereof. However, linking changes in structure in protein mutants to functional impact remains an active area of method development. If successful, such methods can ultimately assist physicians in taking appropriate treatment decisions.”

2) I am not familiar with the genetics of LQTS/LQT1, so it would be great if the authors could comment on whether all the disease variants are dominant, recessive, work via compound heterozygosity or what the genetics is. In particular, I would like them to provide evidence that training a model based on experimental data of homozygous cells is relevant for the disease (in particular since I assume KCNQ1 is a tetramer).

We added following lines in the manuscript to comment on the genetics of LQT1.

Page 5, Lines 97-106:

“KCNQ1 is most often associated with autosomal dominant LQTS, and rarely with recessive LQTS. Dominant-negative loss-of-function (LOF) mechanisms have been implicated in autosomal dominant LQT1. Patients with heterozygous mutations are associated with autosomal dominant LQTS (Romano–Ward syndrome) whereas patients with homozygous or compound heterozygous KCNQ1 mutations have a more severe clinical outcome and are associated with recessive LQTS (Jervell–Lange–Nielsen syndrome)(33). Even for the common heterozygous forms of LQTS, it is valuable to predict the function of a variant in the homozygous state; mainly for discriminating benign from pathogenic variants. This knowledge contributes to determining the disease-causing propensity of variants found in recessive LQTS and identifies variants with potential to cause autosomal dominant LQTS.”

We added Lines 392-403 in the manuscript to provide evidence that training model based on experimental data of homozygous cells is relevant for the disease.

Page 18, Lines 392-403:

*A note on training the ANNs on functional data tested in the homozygous state*

The paper in Science Advances 6, 2018 by Huang et al. provides evidence that training models based on experimental data collected on homozygous cells is relevant for KCNQ1 related diseases (19). Specifically, this group conducted experiments in which WT KCNQ1 was co-expressed with a mutant of interest and the total trafficking of KCNQ1 was quantitated (see Figure 3 in reference 19). They found that the total and cell surface expression of total KCNQ1 (WT + mutant) was usually in between the results for WT only and mutant-only. However, there were a few exceptions in which the WT protein appeared to rescue trafficking of the mutant or where the mutant protein impeded trafficking of the WT protein. These results suggest that studies of mutant-only condition are usually a good predictor for the corresponding WT/mutant heterozygous conditions, but there are exceptions.

Page 25, Lines 540-543:

“Another limitation of this study is that variants were tested in the homozygous state. This work is sufficient for evaluating disease-causing propensity of variants found in severe cases with recessive LQTS but might not address all cases of dominant LQTS.”

3) It would be useful if the authors could explain better both the idea behind their “silent” variants and how they control for that they don’t lead to artificial improvements. I assume the problem is that there are not sufficiently many known benign variants (or variants that have “neutral” phenotypes according to the criteria in Table 1). I couldn’t easily find the numbers, but would be good to know exactly how many “real” “neutral/benign” variants they have. Also, given that many of the features would be zero (I think) for these silent variants, how do the authors know that the ANN isn’t just learning to recognize this? Does the method work as well to recognize real neutral variants as it does for the artificial ones? In this regard, I am curious about the statement: “We observed that introduction of second hidden layer improved the performance of the models on silent mutations with no effect on the prediction accuracy for functional variants.” Ideally, the authors should provide some assessment of accuracy for actual benign variants even if the method is trained on the artificial ones also

The term “silent variant” is reserved typically for SNP gene variations and might be confusing when used in the present context. Thus, we re-named these silent variants as “non-perturbing variants” in our manuscript and in this response letter.

We made appropriate changes to the text and included a plot in the supporting information to depict accuracy of ANN models based on type of variants. To control for artifactual improvement, ANNs were trained by oversampling three functional variants for every non-perturbing variant. Moreover, we report results for benign and pathogenic variants that were not part of the training and monitoring dataset. The method works well in recognizing non-perturbing and neutral variants by predicting the non-perturbing, neutral, and pathogenic variants at different regions as shown in Figure S6.

We added following text in our manuscript to elaborate on the importance of non-perturbing variants in training dataset, and number of variants in our functional dataset.

Page 8, Lines 170-174:

“These non-perturbing variants expose ANNs to all the structural regions of the protein during training. This helps the model to recognize changes in structure and physicochemical properties at the site of mutation for all neighborhoods that exist in the structure. The extent of these changes helps ANNs to classify the phenotype of a mutation (i.e., benign or pathogenic).”

Page 8, Lines 176-178:

“In summary, there were 345 non-perturbing, 39 benign and 86 pathogenic variants based on the peak current criteria given in Table 1.”

We added Lines 334-341 and Figure S6 to depict the accuracy of benign and non-perturbing mutations.

Page 16, Lines 334-341:

“The accuracy of the combined ANN model for non-perturbing, benign, and pathogenic variants is shown in Figure S6. This figure depicts that ANN can recognize these three types of variants and predict majority of them at different regions between 0 and 1. Additionally, to quantify the separation between non-perturbing and benign mutations, we calculated entropy at 0.05 as the decision boundary to separate non-perturbing from benign variants. The lower the entropy (best~0, worst~1), the better is the separation of the two classes. Entropy for all the functional parameters was less than 0.35 suggesting the model can distinguish between non-perturbing and benign variants.”

Figure S6: Distribution of Prediction by ANN for non-perturbing, benign, and pathogenic variants depicting that ANN can distinguish these variants by predicting in three different regions between 0 and 1. Decision threshold is between benign and pathogenic variant

4) If I understand correctly, in their predictions, the authors lump together loss of function and gain of function variants as one class. Is that correct? It would have been useful to know how many are in each class, and how the predictions work for them separately? I would e.g. have guess that the evolution-based methods work better for LOF than for GOF.

Yes, LOF and GOF were grouped together as pathogenic variants. There were 71 LOF and 15 GOF and 39 WT-like variants in our functional dataset. Yes, evolution-based method and rest of the models predict LOF variants better than GOF variants and WT-like variants. We added text and Figure S7 depicting predictive ability of ANN based on type of the variants.

Page 16, Lines 342-348:

“*Predictive ability based on the function of the variants*

Using peak current density to define variant classes, there were 71 LOF, 15 GOF, and 39 WT-like variants in the functional dataset. All ANN models predict LOF better than GOF or WT-like (see Figure S7). Biophysical features-based model predicts GOF better than other feature sets whereas evolutionary-based model predicts LOF better than the rest. By combining biophysical and evolutionary features, there is a significant improvement in predictive ability of WT-like variants.”

Figure S7: Percentage of accurate predictions for GOF, LOF and WT-like based on peak current density by the three ANNs models considered in this study

5) The authors use a relatively simple PSSM-based method to estimate variant effects from the alignments. That’s of course completely valid, but as they are well aware there are plenty of more advanced methods out there that use more information in the alignments. I would recommend that the authors either use or benchmark with these methods or alternatively tone down the fact that the structure/biophysics-based methods are “better”. Perhaps just say that they are better than using a PSSM, but that more advanced sequence-based methods are known to provide better predictions than PSSMs (at least in many cases).

We toned down our argument by changing the wording as ‘biophysical features are better than PSSM-based evolutionary features or PSSM amino acid substitution scores’ to present a clear picture to our audience. We have made these changes throughout our manuscript.

6) The authors write “MCC values were computed by using Biochemical Library (BCL) wherein the threshold was adjusted individually for IKs, V1/2, τact and τdeact from 0 to 1 to achieve the best MCC for each phenotype.”. How precisely were these hyper-parameters chosen and did the authors do anything to avoid overfitting?

The decision boundary was chosen based on the optimal MCC achieved for each parameter. We added Lines 614-615 to comment on the fluctuation of the decision boundary.

Page 28, Lines 614-615:

“This hyper-parameter fluctuated roughly 20% based on different instances of the model.”

The introduction of drop out in the input and hidden layers help ANN models to avoid overfitting. We added Lines 575-578 to address this point.

Page 26, Lines 575-578:

“ANNs were trained with dropouts to prevent overfitting. For instance, the introduction of dropouts in the input layer and hidden layers improve average MCC for the biophysical model from 0.38 to 0.49.”

7) One of the main points from the authors is that the predictions based on biophysical features can be used to gain mechanistic insight. It would this be useful and interesting with some examples of this. The input-sensitivity analysis provides a bit of this, but it’s not really very detailed. This is complicated further by the fact that many of these scores may be correlated with each other, and given that they use relatively many features could make it difficult to pinpoint specific effects. That said, I think it would be relevant if the authors could make this part a bit clearer and perhaps discuss some examples of variants that they think they can explain why cause LOF.

We agree with the reviewer that examples of variants can provide better clarity to gain mechanistic insight. Thus, we have added variant specific examples to support input sensitivity analysis and highlight significance of our biophysical features. The following lines has been added to the manuscript.

Page 20, Lines 423-428:

For instance, at R195 the functional density of polarizability is 0.19 for radius size of 6.5 Å. Mutation at this site to Q and P decreases site polarizability. R195Q and R195P exhibit LOF possibly because changed amino acid polarizability affects KCNQ1 activation. This is in line with the high sensitivity of V_1/2_ prediction for polarizability as shown in Figure 5. Similarly, R195W is a GOF mutant and this correlates with increase in the site polarizability.

Page 20, Lines 429-438:

L114P, E115G, Y125D, G189A, S225L, L236P, and L236R are variants that fail to activate due to protein misfolding(19). These variants have similar native hydrophobicity and neighborhood hydrophobicity, however, mutations at these sites result in changes in hydrophobicity suggesting that the amino acid side chains for these variants are located in an energetically unfavorable environment. Therefore, network predictions have high sensitivity for mutant hydrophobicity, and functional density for hydrophobicity, suggesting that protein stability is a crucial aspect in interpreting the functional phenotype of V_1/2_ and 𝜏_deact_. Exposure of a site (neighbor vector) to solvent environment is also sensitive to functional phenotype of V_1/2_ and 𝜏_deact_.

Page 21, Lines 465-471:

“For example, variants R259H, V280E, A300E, A300T, F340L, A344V and others are located in membrane-embedded regions of KCNQ1 and near to the pore domain cause LOF. On the other hand, site A287 is in close proximity to the pore but lies outside the membrane. This could explain why A287E and A287V variants are WT-like for all functional parameters. Proximity to the pore and location in the membrane appear to classify sites that are in functionally critical regions of the protein.”

Page 23, Lines 511-513:

“Interestingly, the majority of the sites such as R116, R195, K196, Y184, K183, R181, and R249 are located within 4 Å distance from the PIP_2_ interacting sites at the S2-S3 linker, S3 helix, S4 helix, and S4-S5 linker that are proton donors and have high polarizability.”

8) I thank the authors for making a web-server available. That’s great. I would recommend that they also make their code available so that others in principle can look underneath the hood. Also, it would be good to store the multiple sequence alignment that is used to calculate the PSSM.

BCL is publicly available for academic use. We have captured our protocol in the file “Protocol Capture.docx”. All the methods and features used have been cited and mentioned in detail in the supplement material. The files ‘a1q1.uniref50.0.00001.ascii4’ and ‘a1q1.nr.ascii4’ in the compressed zip file “protocol files” provide multiple sequence alignment PSSM matrices for the 676 KCNQ1 sites that were used for calculating PSSM scores.

Minor:

1) p. 4, line 70: The authors write “membrane proteins” but refer only to data for KCNQ1 in LQT1. I suggest either making it clear that it’s only a single protein or provide references for other proteins whose destabilization and mistrafficking is involved in LQT1.

We acknowledge the concern and append the text in the manuscript as following.

Page 4, Line 76-78:

“This observation underscores the growing notion that mutation-induced destabilization and mis-trafficking of the KCNQ1 protein are common disease mechanisms in LQT1.”

2) p. 4, line 81–82: The authors write “but these tools have limited applicability for KCNQ1”. Could they provide a citation or evidence for this?

We have provided a citation for our statement.

Page 5, Line 88-89:

“but these tools have limited applicability for KCNQ1(see Table S4 in reference 22)”

3) p. 5, line 99: I’ll let it be completely up to the authors, but I would recommend deletion of “advanced and innovative”

We removed the adjectives referring to the KCNQ1 predictor.

Page 6, Line 118-119:

“In this study, we used machine learning to develop a KCNQ1 variant prediction tool called Q1VarPredBio”

4) p. 13: The authors write “We found that three out of 14 features used for biophysical model failed to improve the predictions on the unseen dataset, therefore were excluded”, but I couldn’t see this analyses

We changed the text and introduced a plot to support our argument (Figure S8).

Page 14, Line 289-293:

“We found that three [Neighbor Vector, Mutant steric parameter, and Native steric parameter] out of 14 features used for the biophysical model and uniref50 based PSSM scores failed to improve the predictions on the unseen dataset, therefore these features were excluded (see Figure S8). This could be due to redundancy in the information carried by these three biophysical and evolutionary features.”

Figure S8: Exclusion of 3 biophysical features and 1 evolutionary feature does not affect the performance when evolutionary and biophysical features are combined

Reviewer #2:

In the present manuscript, the authors used machine learning to develop an advanced method for functional prediction of KCNQ1 mutants. The functional classification categories of this method were then expanded by a scheme that was able to predict variant-specific changes in four electrophysiological KCNQ1 parameters (namely peak current density, voltage of half-maximal activation, and activation and deactivation time constants). The authors then evaluated the performance of artificial neural networks trained on evolutionary and biophysical features for KCNQ1 and observed that a combination of both features produced a model with good predictive accuracy. This study is complementary to several previous studies in which artificial intelligence was used to predict protein structure and the properties of its mutants. The concept behind this paper have merit. In fact, this concept is intriguing and has the potential to provide important new information for both basic (mode of action) and applied science (therapy). Although this manuscript is original and novel, one major issue is that in this study, only a single type of protein is being tested as a case study which questions the generalization of the results. The authors should therefore test an additional type of protein to make the results and conclusions more universal. In addition, the authors trained and also validated their models with 125 KCNQ1 variants that were functionally evaluated by others; there is thus a need for more functional data – for example, for variants within different domains of the protein. The authors should test/validate their prediction using mutants that are not included in the training sets. In general, I think that the computational experiments and analysis described are developed sufficiently within the manuscript to inspire confidence that the findings produce meaningful results, and as mentioned above, this paper describes an interesting concept but the fact that this analysis was done for only a single type of protein and that training and validation were performed on the same variants are a major issues and must be addressed before acceptance of this paper for publication.

We acknowledge the concern raised by the reviewer related to generalizability of this method. This study was enabled by functional data available for KCNQ1 sufficiently abundant to train a neural network. The lack of sufficient amount of functional data collected under similar experimental protocols for other proteins makes it challenging to train and test our method on other ion channel proteins. Moreover, the main idea of the paper is to seek biophysical features that perform as good as evolutionary features. Thus, testing out the models on another protein is out of scope. We also note that type 1 long QT syndrome is among the most common genetic disorders, afflicting on the order of 1:2500 human (see reference 3), often causing sudden death. We respectfully argue that promising new approaches to predict the possible pathogenicity of KCNQ1 mutations are of high significance even if they cannot immediately be extended to other protein/disease relationships.

All ANN models were tested using variants that were not part of the training dataset. Out of 470 variants, roughly 19 variants were used as a prediction dataset and another 19 variants as a monitoring dataset. All the models were trained on 432 variants, and predictions were made on 19 unseen variants. We randomly shuffled our data 330 times and utilized 25 cross validation technique to remove any bias. All the results reported in this manuscript were on unseen dataset. Lines 594-597 around cross validation was revised to bring more clarity.

Page 27, lines 594-597:

“For better generalizability and balancing the different classes within the training, monitoring, and independent subsets, we adopted a 25-fold cross-validations strategy, wherein 23 subsets (typically 432 variants) were utilized for training, one subset for monitoring (19 variants), and one subset for prediction (19 variants).”
